# King Edward's Hospital Fund for London

**Published:** 1913-12-20

**Authors:** 


### KING EDWARD'S HOSPITAL FUND FOR LONDON.

### Annual Meeting to Award Grants.

A meeting of the Governors and General Council of King Edward's Hospital Fund for London for the purpose of awarding grants to the hospitals, convalescent homes, and consumption sanatoria for the present year was held on the 12th inst. at St. James's Palace, his Highness the Duke of Teck being in the chair.

Those present were the Speaker of the House of Commons (the Right Hon. James W. Lowther, M.P.), the Rev. W. L. Watkinson, D.D., the Chief Rabbi (the Rev. Dr. J. H. Hertz), Lord Rothschild, Lord Stamfordham, the President of the Hospital Sunday and Hospital Saturday Funds (the Right Hon. the Lord Mayor), the Governor of the Bank of England (Mr. Walter Cunliffe), the President of the Royal College of Physicians (Sir Thomas Barlow, Bart.), the President of the Royal College of Surgeons (Sir Rickman J. Godlee, Bart.), the Right Hon. Sir Savile Crossley, Bart. (Hon. Secretary), the Right Hon. Sir Vezey Strong, Sir William Church, Bart., Sir Henry Burdett, Sir Horace Marshall, Sir Cooper Perry, Sir John Craggs, Sir John Tweedy, Mr. Frederick M. Fry, Dr. Edwin Freshfield, Mr. John G. Griffiths (Hon. Secretary), and Mr. Algernon E. Sydney.

### The Wernher Windfall of £465,000.

Lord Rothschild (the Hon. Treasurer), in reporting that the amount received by the Fund to the 6th inst., after payment of expenses, was £150,692, said the Fund had the sum of £170,000 on deposit with Messrs. Baring Brothers, who had allowed upon this sum a very generous rate of interest, for which the subscribers ought to be very grateful. He had a very pleasant piece of information to give to the Council which he had received from Mr. Eckstein, one of the executors of the late Sir Julius Wernher. Sir Julius Wernher had been a member of this Council, and had taken a great interest in its affairs; and besides making a specific legacy in his will he left this Fund one-twelfth of his residuary estate. A sum of £265,000 would shortly be received, to be followed later on by a further amount of probably £200,000, making £465,000 in all. In connection with this large windfall and also with the moneys received and to be received from the late Mr. Samuel Lewis's estate, he proposed that these sums be invested permanently, and not treated in the same way as ordinary legacies, because the Fund could not look forward to receiving such large amounts very often. He moved that a resolution of thanks be sent to Mr. Eckstein for the great trouble he had taken in winding up the estate.

The vote of thanks was seconded by his Highness the Duke of Teck, and carried unanimously.

### The League of Mercy.

Sir Henry Burdett, in making his annual statement on behalf of the League of Mercy, said it had been stated lately in the press that the whole of the hospitals were suffering very much this year; and there could be no doubt that, owing to recent legislation, the year 1913 had been, and would be, the trial year of the voluntary system of hospitals in this country. He had given a great deal of attention to the effects and the probable result, and he was glad to say that on the figures, as far as they were now available, the voluntary principle had triumphed, that it was deep-seated in the hearts of the English people, and that the voluntary hospitals were certainly safe, in the financial sense, on their present basis. (Hear, hear.) So far as the League of Mercy had yet received returns from the different districts, the amounts that would be received were practically identical with those of last year, and they would certainly bring the total contributions from the League of Mercy to the King's Fund, by the end of the present year, up to a sum considerably exceeding £200,000.

### The Distribution Committee's Report.

Sir William Church (the Chairman of the Distribution Committee) read the report of that committee as follows:—

The Governors and General Council having again placed the sum of £151,000 in the hands of the committee for distribution amongst the London hospitals, the committee beg to report as follows:—

(1) The number of hospitals applying for grants is 106, as against 104 last year.

(2) Although the total amount to be distributed remains the same, the grants recommended continue to show, as compared with previous years, numerous and in some cases considerable variations. This is due to the fact that the grants are based on an examination of all the circumstances affecting the claims of each hospital each year, in relation to those of other hospitals, including the financial position of each institution as disclosed by its income and expenditure account and balance sheet for the last completed year. It must not be assumed, therefore, that a reduction in the maintenance grant of any particular institution, any more than the absence of a grant, implies dissatisfaction.

(3) The Mount Vernon Hospital for Consumption does not now appear in the list, since the hospital at Hampstead has not applied, and the hospital at Northwood, being no longer a branch of a hospital applying for a grant, and being itself outside the area dealt with by the Distribution Committee, does not come within the scope of this Report.

(4) The committee are glad to report that the authorities of St. George's Hospital have rendered their accounts for the year 1912 in an amended form, which reduces the amount paid to the Medical School out of the general fund of the hospital, in respect of specific work done for the patients, to a sum not exceeding that which was charged prior to the changes which led to the suspension of the Fund's grants. The committee, therefore, find themselves in a position to recommend a grant to St. George's Hospital, the amount of which is based on a consideration, on their merits, of the claims of the hospital in respect of the work of the present year. The grant will be subject to a certificate from the chairman of the hospital that the accounts for 1913 will be in conformity with those now presented for 1912. The position will then be that, although St. George's received a grant in 1909, for which year its accounts when made up and published in the spring of 1910 proved to be in an incorrect form, it forfeited grants not only in 1910 and 1911, when the accounts were in the same incorrect form, but also in 1912, for which year the accounts have now been submitted in the correct form in which they were rendered prior to 1909. Under these circumstances the conditions set out in the Fund's rule and in the previous Reports of the committee will be fully satisfied by the present recommendation.

(5) The committee are glad to learn that further progress has been made with the proposal for the amalgamation of the British Lying-in Hospital and the Home for Mothers and Babies at Woolwich. The committee recommend that a grant of £1,500 be set aside this year towards this amalgamation, subject to the scheme, when mature, being submitted and approved.

(6) It was decided in 1911 that the grants promised or set aside towards a general amalgamation of the five Throat, Nose, and Ear Hospitals should remain at the figure of £20,500, pending an agreement on a satisfactory scheme or schemes. The committee are informed that a partial scheme is at present under consideration.

(7) The committee view with great satisfaction the opening of the new King's College Hospital in South London. They recommend a further grant of £5,000 to the Removal Fund, thus raising the amount contributed by the King's Fund to this object to £53,000.

(8) The Reports of the Visitors bear witness, as usual, to the care and thoroughness with which the annual inspections are carried out by the medical men and laymen who undertake the work. The committee have for many years made a practice of accompanying the Fund's grants, where desirable, by observations extracted from these Reports, and they have found, as the result of inquiry by the Visitors of the following year, that the suggestions thus brought to the notice of the hospital authorities have often proved of practical value.

(9) The committee have also accompanied the grants in some cases with remarks arising out of their own examination of the circumstances of particular hospitals. In one or two instances they have recommended that these remarks should be made public.

(10) As in former years, many special grants are recommended in aid of schemes of capital expenditure, particulars of which had previously been submitted to the Fund and examined by the committee.

(11) The committee desire to draw the attention of the promoters of new hospitals to the fact that the invitation to submit their proposals to the Fund, originally issued by the General Council in 1903 and since repeated from time to time, was addressed to them as well as to hospitals reconstructing or extending. During the past two years the committee have been consulted upon three new schemes. It is not the practice of the Fund to entertain applications for grants from any institution that has not been in existence for at least three years. But it is obviously preferable, in the interests of any proposed new institution as well as on general grounds, that the opinion of the Fund on the question of the necessity for a new hospital should be sought before, rather than after, any definite action is taken.

(12) In view of the general increase in hospital expenditure shown by the Fund's Statistical Report for 1912, the committee made special inquiries into all cases where there had been any considerable rise in cost, so as to be in a position to distinguish between increases due to unavoidable causes, or to circumstances common to hospitals generally, and increases which it might be within the power of the managers of the individual hospitals to control. Where it seems desirable, attention is privately drawn to the excess of cost in a remark attached to the grant.

The details of the awards to the various hospitals are appended.

### Grants Recommended by the Distribution Committee.

*The Distribution Committee desire to draw attention to the fact that it must not be assumed that the absence of a grant implies dissatisfaction.*

Alexandra Hospital for Children, £250; of which £50 to balconies.

Beckenham Cottage Hospital, £25. Belgrave Hospital, £1,250; of which £500 to reduce building debt. Blackheath and Charlton Cottage Hospital, £150; of which £50 to new Nurses' Home. Bolingbroke Hospital, £1,000. British Lying-in Hospital; hospital in Endell Street closed; a grant of £1,500 is set aside towards amalgamation with the Home for Mothers and Babies, Woolwich, in accordance with scheme to be approved (see paragraph 5 of Report).

Central London Ophthalmic Hospital, £750; of which £500 to equipment of new building. Central London Throat and Ear Hospital; capital grants amounting to £20,500 have been set aside and promised for the purpose of a general amalgamation of the Throat, Nose, and Ear Hospitals (see paragraph 6 of Report). Charing Cross Hospital, £5,500; of which £1,000 to redemption of mortgage debt and £1,500 to new free wards in accordance with the scheme submitted to the Fund. Chelsea Hospital for Women, £2,000; of which £1,500 to rebuilding in accordance with the scheme submitted to the Fund. Cheyne Hospital for Sick and Incurable Children, £50; in consideration of the fact that some curable cases are admitted. City of London Hospital for Diseases of the Chest, Victoria Park, £4,000; of which £500 to improvements, subject to scheme being submitted to the Fund. City of London Lying-in Hospital, £500. Clapham Maternity Hospital, £2,100; of which £2,000 to reconstruction in accordance with the scheme submitted to the Fund.

"Dreadnought" Hospital, £4,000; of which £500 to renewal of floors.

East End Mothers' Lying-in Home, £200. East London Hospital for Children, £2,000. Eltham and Mottingham Cottage Hospital, £75.

Finchley Cottage Hospital, £25. Florence Nightingale Hospital for Gentlewomen, £200. French Hospital, £100. Friedenheim Hospital, £75.

General Lying-in Hospital, £200. German Hospital, £100. Gordon Hospital for Fistula, etc., £25. Great Northern Central Hospital, £5,000; of which £2,000 to reduce debt on general fund. Grosvenor Hospital for Women, £350. Guy's Hospital, £7,000; of which £1,500 to building improvements in accordance with the scheme submitted to the Fund.

Hampstead General Hospital, £2,750; of which £500 to reduce general fund debt and £500 in accordance with agreement upon amalgamation with North-West London Hospital. Home and Infirmary for Sick Children, £250. Home for Mothers and Babies, Woolwich, £150; to maintenance; a grant of £1,500 is set aside towards amalgamation with the British Lying-in Hospital in accordance with scheme to be approved (see paragraph 5 of Report). Hornsey Cottage Hospital, £25. Hospital and Home for Incurable Children, Hampstead, £50; in consideration of the fact that some curable cases are admitted. Hospital for Consumption, Brompton (including Sanatorium at Frimley), £4,500. Hospital for Diseases of the Throat; capital grants amounting to £20,500 have been set aside and promised for the purpose of a general amalgamation of the Throat, Nose, and Ear Hospitals (see paragraph 6 of Report). Hospital for Epilepsy and Paralysis, £1,500; of which £500 to recent extension in accordance with the scheme submitted to the Fund. Hospital for Sick Children, £3,000. Hospital for Women, Soho Square, £2,000; of which £1,000 to reduce mortgage debt. Hospital of St. John and St. Elizabeth, £400.

Infants' Hospital, £25. Italian Hospital, £200.

Kensington and Fulham General Hospital, £25. Kensington Dispensary and Children's Hospital, £25. King Edward Memorial Hospital (Ealing), £550; of which £150

to provision of mortuary. King's College Hospital, £6,500; of which £5,000 to Removal Fund.

Leyton, Walthamstow, and Wanstead Children's and General Hospital, £350. London Fever Hospital, £2,050; of which £2,000 to rebuilding of Isolation Block in accordance with the scheme submitted to the Fund. London Homœopathic Hospital, £600. London Hospital, £12,000. London Lock Hospital, £3,250; of which £1,100 to the Harrow Road Hospital, £500 to sanitary improvements at the Harrow Road Hospital, and £1,650 to enable the new hospital at Dean Street to be equipped and opened free of debt. London Temperance Hospital, £1,200. London Throat Hospital; capital grants amounting to £50,500 have been set aside and promised for the purpose of a general amalgamation of the Throat, Nose, and Ear Hospitals (see paragraph 6 of Report).

Maternity Charity and District Nurses' Home, Plaistow, £200. Medical Mission Hospital, Balaam Street, Plaistow, £350. Memorial Hospital, Mildmay Park, £50. Metropolitan Hospital, £3,000. Middlesex Hospital, £5,000; of which £500 to improvements. Middlesex Hospital Cancer Charity, £100. Mildmay Mission Hospital, £400; of which £100 to scheme for new lift. Miller General Hospital for South-East London, £2,500; of which £1,500 to extension in accordance with the scheme submitted to the Fund.

National Dental Hospital, £300. National Hospital for Diseases of the Heart. National Hospital for the Paralysed and Epileptic, £3,000; of which £500 to reduce debt. Nelson Hospital (late South Wimbledon, etc.), £400; of which £200 towards completion of building. New Hospital for Women, £500. Norwood Cottage Hospital, £25.

Paddington Green Children's Hospital, £500. Passmore Edwards Acton Cottage Hospital, £150. Passmore Edwards East Ham Hospital, £75. Passmore Edwards Hospital for Willesden, £25. Passmore Edwards Cottage Hospital, Wood Green, £50. Poplar Hospital, £400; of which £200 to new lift in accordance with the scheme submitted to the Fund. Prince of Wales's General Hospital, £4,500; of which £2,000 to reduce debt; the committee are glad to observe the economical administration of this hospital, which they consider should receive more substantial support from the public.

Queen Charlotte's Lying-in Hospital, £1,750; of which £1,000 to new out-patient department in accordance with the scheme submitted to the Fund. Queen's Hospital for Children, £3,250; of which £1,250 to reduce building debt.

Royal Dental Hospital of London, £750; of which £500 to reduce building debt. Royal Ear Hospital; capital grants amounting to £20,500 have been set aside and promised for the purpose of a general amalgamation of the Throat, Nose, and Ear Hospitals (see paragraph 6 of Report). Royal Eye Hospital, £500. Royal Free Hospital, £5,000; of which £2,500 to rebuilding of out-patient department in accordance with the scheme submitted to the Fund. Royal Hospital for Diseases of the Chest, City Road, £1,250. Royal Hospital, Richmond, £200. Royal London Ophthalmic Hospital, £2,250. Royal National Orthopædic Hospital, £3,000; of which £1,000 to reduce debt. Royal Waterloo Hospital for Children and Women, £1,250. Royal Westminster Ophthalmic Hospital, £50.

St. George's Hospital, £3,000. St. John's Hospital for Diseases of the Skin. St. John's Hospital, Lewisham, £300 St. Luke's House, £25. St. Mark's Hospital, £400. St. Mary's Hospital, £5,500; of which £500 to reduction of general fund debt, and £500 to reconstruction of Casualty Department in accordance with the scheme submitted to the Fund. St. Mary's Hospital for Women and Children, Plaistow, £2,000; of which £1,500 to new Nurses' Home and out-patient department, subject to the scheme being submitted to the Fund. St. Monica's Hospital, £50. St. Peter's Hospital for Stone, £150. St. Saviour's Hospital, Osnaburgh Street, £150. Salvation Army Maternity Hospital; the new hospital was only opened at the end of October. Samaritan Free Hospital, £1,000. Santa Claus Home, £25.

University College Hospital, £4,000.

Victoria Hospital for Children, £800.

West End Hospital for Diseases of the Nervous System, £350. Western Ophthalmic Hospital, £500; to reduction of debt on rebuilding of out-patient department. West Ham and East London Hospital, £3,500; of which £500 to recent improvements. West London Hospital, £5,000; of which £2,000 to new Nurses' Home, subject to the scheme being submitted to the Fund. Westminster Hospital, £3,500; of which £500 to reduce debt. Wimbledon Cottage Hospital, £100. Woolwich and Plumstead Cottage Hospital, £50.

Total ... ... ... ... ... £149,500.

SPECIAL GRANT.—Set aside for amalgamation of British Lying-in Hospital and the Home for Mothers and Babies, Woolwich, £1,500.

SUMMARY.

| | |
|---|---|
| Grants ... ... ... ... ... ... ... | £149,500 |
| Special Grant set aside for amalgamation ... | 1,500 |
| **Total Grants to Hospitals for the year 1913** | **£151,000** |

### The Convalescent Homes Committee's Report.

In the absence of Mr. William Latham, K.C. (the Chairman of the Convalescent Homes Committee), Dr. Edwin Freshfield read the Report of that committee as follows:—

(1) The Governors and General Council have this year again placed the sum of £6,500 in the hands of the committee with a view to distribution amongst convalescent homes and consumption sanatoria.

(2) The number of applications eligible for consideration amounted to 46 from convalescent homes and 10 from consumption sanatoria, as against 44 and 10 respectively last year.

(3) In dealing with applications from convalescent homes the committee have adhered to their original policy of giving special consideration to the convalescent homes closely connected with London hospitals.

(4) The committee have continued the practice of making grants to consumption sanatoria on the basis of the reservation of a certain number of beds for the use of patients from London hospitals. The accommodation thus secured this year consists of 44 beds at five sanatoria, as against 39 beds at five sanatoria last year, and is situated as follows:—

Two beds for children at the Maitland Sanatorium, Peppard, Oxon; ten beds at the Devon and Cornwall Sanatorium, South Brent, Devon; ten beds at the Kelling Sanatorium, Holt, Norfolk; ten beds at the Sanatorium of the National Association for the Establishment and Maintenance of Sanatoria for Workers, Benenden, Kent; twelve beds at the Northampton Sanatorium, Creaton, Northants.

(5) In allocating these beds to hospitals in London the committee have followed the same principle as that adopted last year, viz.: that preference should be given (in order of relative size) to the two consumption hospitals which have no country branches and that the rest of the beds should be divided among the largest of the general hospitals which apply to the Fund.

They accordingly recommend the following allocation:—

Eighteen beds to the City of London Hospital for Diseases of the Chest; nine beds to the Royal Hospital for Diseases of the Chest; six beds to the London Hospital; six beds to Guy's Hospital; five beds to the Middlesex Hospital.

(6) The committee made inquiries both from the sanatoria and from the London hospitals to ascertain whether the supply of beds for consumptive patients was affected by the working of the National Insurance Act. The result of these inquiries indicates that, for the present at all events, the accommodation provided for tuberculosis patients in accordance with the Act has not been sufficient to reduce in any appreciable degree the need for the grants made to sanatoria by the Fund.

(7) All the sanatoria applying for grants have been inspected on behalf of the Fund, and the committee desire to express their indebtedness to the Visitors who undertook the necessary journeys.

(8) The grants recommended to the various institutions are appended.

### Grants Recommended by the Convalescent Homes Committee.

A.—Consumption Sanatoria.

*The Convalescent Homes Committee desire to draw attention to the fact that it must not be assumed that the absence of a grant implies dissatisfaction.*

Children's Sanatorium, Holt, Norfolk, £50. Daneswood Sanatorium (Jewish), Woburn Sands, £25. Devon and Cornwall Sanatorium, Didworthy, South Brent, £875; in consideration of the reservation of ten beds during 1914 for the use of certain London hospitals (see paragraphs 4 and 5 of Report). Eversfield Chest Hospital, St. Leonards, £50; towards the provision of a fire-escape staircase. Fairlight Sanatorium, Hastings, £50. Kelling Sanatorium, Holt, Norfolk, £875; in consideration of the reservation of ten beds during 1914 for the use of certain London hospitals (see paragraphs 4 and 5 of Report). Maitland Sanatorium, Peppard, Oxon, £125; in consideration of the reservation of two children's beds during 1914 for the use of certain London hospitals (see paragraphs 4 and 5 of Report). Mount Vernon Hospital, Northwood. National Association for the Establishment and Maintenance of Sanatoria for Workers, Benenden, Kent, £875; in consideration of the reservation of ten beds during 1914 for the use of certain London hospitals (see paragraphs 4 and 5 of Report). Northampton Sanatorium, Creaton, Northants, £1,050; in consideration of the reservation of twelve beds during 1914 for the use of certain London hospitals (see paragraphs 4 and 5 of Report).

Total ... ... ... ... ... ... £3,975

B.—Convalescent Homes.

*The names of Convalescent Homes receiving no grant are not published.*

Alexandra Hospital for Children, Painswick, £25. Baldwin Brown Home for Convalescent Poor, Herne Bay, £25. Beau Site Convalescent Home, Hastings, £25. Brentwood Convalescent Home for London Children, Brentwood, £25. Catherine Gladstone Free Convalescent Home for the Poor, Mitcham, £50. Charing Cross Hospital, Limpsfield, £12[illegible] with a view to encouraging the admission without payment of patients direct from the hospital. Chelsea Hospital for Women, St. Leonards, £50; with a view to encouraging the admission without payment of patients direct from the hospital. Children's Cottage Hospital, Cold Ash, Newbury, £25; in consideration of convalescent cases. Children's Home Hospital, Barnet, £25; in consideration of convalescent cases. Convalescent Home for Poor Children, St. Leonards, £25. Convalescent Police Seaside Home, Hove, £25. Deptford Medical Mission Convalescent Home, Bexhill, £25. Erskine House, Hampstead Heath, £25. Friendly Societies' Convalescent Homes, Dover and Herne Bay, £50. Great Northern Central Hospital, Clacton-on-Sea, £125. Highgate Convalescent Home for Children (formerly All Saints' Home), Highgate, £25. Home and Infirmary for Sick Children, Sydenham, Broadstairs, £50. Hospital for Sick Children, Great Ormond Street, Highgate, £100. King's College Hospital, Hemel Hempstead, £100. London Hospital Convalescent Home, Tankerton, £75. Mary Wardell Convalescent Home for Scarlet Fever, Stanmore, £25. Metropolitan Convalescent Institution, Walton, £150. Middlesex Hospital, Clacton, £400. Mrs. Kitto's Free Convalescent Home, Reigate, £75. National Hospital for the Paralysed, Finchley, £100; with a view to facilitating the admission of patients without payment. Paddington Green Children's Hospital, Slough, £100. Poplar Hospital, Walton-on-Naze, £50. Queen Charlotte's Lying-in Hospital, Kilburn, £50. Queen's Hospital for Children, Bexhill, £125. St. Andrew's Convalescent Home, Folkestone, £50. St. Andrew's Convalescent Hospital, Clewer, £25. St. Mary's Home for Children, Broadstairs, £50; in consideration of convalescent cases. St. Michael's Home for Men and Women, Westgate-on-Sea, £25. Sunshine Home of Recovery, Hurstpierpoint, £25. Surgical Home for Boys, Banstead, £50. Tilford Convalescent Home for Children, Farnham, £25; to building improvements. Victoria Home for Invalid Children, Margate, £25; in consideration of convalescent cases. Victoria Hospital for Children, Chelsea, Broadstairs, £125. Victoria Hospital for Children, Chelsea, Biggin Hill, Kent, £25. Winifred House Invalid Children's Convalescent Hospital Home, Tollington Park, £25.

Total ... ... ... ... ... ... £2,525

| | |
|---|---|
| Grants to Consumption Sanatoria ... ... ... ... | £3,975 |
| Grants to Convalescent Homes ... ... ... ... ... | 2,525 |
| Total Grants recommended to Convalescent Homes and Sanatoria for the year 1913 ... ... ... | £6,500 |

### The Duke of Teck's Speech.

Congratulations from the King.

His Highness the Duke of Teck, in moving the adoption of the Report and Awards, said :

My Lords and Gentlemen,—On behalf of the Governors I rise to move the adoption of the Reports which have just been read.

Before doing so I have the honour to read the following letter addressed to the Governors by Lord Stamfordham by direction of his Majesty the King, Patron and past President of the Fund :—

"Buckingham Palace,
"December 7, 1913.

"Sir,—I am commanded to express to you and through you to the Council at their meeting on the 12th inst. the congratulations of the King upon the results achieved this year by the Fund.

"There has been an increase in the income from investments, and also to a slight degree in annual subscriptions. Consequently the amount available for distribution is rather larger than last year, and the same sum can be distributed as in 1912 without again drawing on reserves. His Majesty realises that with the rise of prices and the increased cost of special treatments there must be a constant tendency for the needs of the hospitals to become greater. At the same time, grants have been given to two more hospitals this year. For these reasons it is hoped that means may be forthcoming for an extended distribution in the future.

"The King, in following the work of the Fund, fully recognises how much is due to the ungrudging services of the members of the Council and the Standing Committees, and to the never-failing counsel and valuable assistance of the Honorary Secretaries.

"His Majesty had learnt with regret of the retirement, through ill-health, of Sir Trevor Lawrence, one of the oldest members of the Fund and of the Distribution Committee, and also that the Fund is losing Mr. Danvers Power, who was Honorary Secretary from 1904 to 1907, and who initiated the statistical report, and devoted himself for many years to the work of the Fund.

"The King hears with the utmost satisfaction of the able and cordial manner in which the ever-increasing work of the office is conducted under the direction of Mr. Maynard and his staff.

"Your obedient servant,

"The Presiding Governor, "Stamfordham.

"King Edward's Hospital Fund for London."

I am sure you will all desire the Governors, on behalf of themselves and of the whole Council, to tender to his Majesty our humble thanks for his gracious message and for the continued warm interest which he has ever manifested in the working of the Fund founded by his father, King Edward.

It is with a great sense of relief and gratification that we find ourselves able this year to maintain our annual distribution at the high level of £157,500, first attained in 1911. Last year, as you will remember, we suffered from a very serious falling off in the amount received from legacies, and were compelled not only to check the steady rise in the amount distributed, but to draw a considerable sum from our reserves in order to avoid an actual retrogression. We are, fortunately, in a better position to-day. It is true that our receipts from donations—always a fluctuating source of income—are at present rather less than last year. But annual subscriptions show a small increase on last year's estimates, and the estimated income from investments is up by nearly £2,000—the capital receipts from legacies last year having exceeded the amount taken from reserve. Legacies, moreover, are once more up to the average for the past five years—the average upon which the distribution has for some time been roughly based. We are thus able to distribute the same amount as last year, while keeping a small balance in hand—a balance which has been increased by further legacies received since the estimates were drawn up. I am sure you will agree that we are wise in keeping this balance and not at once increasing the distribution. For the effect of the small legacy receipts of last year on the five years' average will be felt for some time, and we shall have to be prudent in applying our funds as well as energetic in trying to increase them, if we are to maintain the distribution at £157,500. We must not forget, or let the public forget, the words of his Majesty, the patron of the Fund, last year, when he said that he would look upon any reduction in the total grants as a calamity. I hope the increase in annual subscriptions, in which, I am glad to say, that members of the Council and of the committees of the Fund themselves took the lead, may be taken as a sign that the public do not mean to allow that calamity to occur.

"We Welcome St. George's Hospital Back."

The Distribution Committee remind us in their Report that even when the total amount distributed remains the same, the individual grants vary from year to year; and they point out that this is because the distribution is carefully adjusted to the temporary variations in the comparative needs of the separate hospitals. For while the needs of the hospitals, taken as a whole, show a tendency to increase, owing largely to the increasing demands for more numerous and more costly special treatments, the finances of individual hospitals have, from year to year, their separate ups and downs. And it is one of the advantages of a Fund like this that it makes a detailed study of the accounts of each hospital, and can vary its awards so as to make up to some extent for these more or less accidental fluctuations.

I am sure we all welcome St. George's Hospital back to our list of grants. I hope it has always been understood by the public that we have never suggested any want of good faith on the part of the hospital authorities. The difficulty only arose because the Fund is bound by a special trust in the matter of the financial relations of hospitals and medical schools. It is quite natural that those who have not the responsibility of carrying out that trust, and have not had the long experience that we have had, in applying the trust impartially to the different circumstances of many different hospitals, should sometimes fail to realise the standpoint from which we are bound to approach any particular case.

We are glad to see that the Convalescent Homes Committee have been able to secure more beds at consumption sanatoria and to include one more hospital in the list of those to whom beds are allotted. Whatever the future may bring forth, in the way of public machinery for dealing with tuberculosis, there is no doubt that at present the demands on the voluntary hospitals for the treatment of consumption in its early stages are most urgent and pressing.

The Fund and Radium.

The Executive Committee have recently had before them a suggestion made by my brother, Prince Alexander of Teck, Chairman of the Middlesex Hospital, that the Fund should purchase a supply of radium, which should be available for the use of the London hospitals through the agency of the laboratories attached to the Middlesex. While fully realising the need of the hospitals for a larger supply of this very valuable and very costly curative agent, the Executive were compelled to reply that, as at present advised, the Fund had no legal power, under its Act of Incorporation, to use its general funds in the purchase of radium in the manner suggested.

I should like to emphasise the remark of the Distribution Committee drawing the attention of the promoters of new hospitals to the invitation of this Council to submit their proposals to the Fund. It is obvious that we cannot communicate with them individually as we can with existing hospitals. We can only issue the invitation as publicly as possible, in the hope that it may become widely known, and that the advantage of seeking the opinion of the Fund at an early stage may become generally recognised.

The statistical report this year again shows an increase in hospital expenditure. The Report recognises that this is in many cases due to causes common to all hospitals, such as the rise in prices and the increase in special or costly treatments. The Distribution Committee have, therefore, acted justly as well as wisely in making special inquiries before drawing attention to high cost at any particular hospital.

The comparisons of cost per bed depend for their accuracy very largely on the degree of uniformity with which the accounts and statistics of the various hospitals are made up. For this uniformity we are indebted to the uniform system of hospital accounts introduced by Sir Henry Burdett, and to the revision of that system adopted by the three Funds—the Sunday Fund, the Saturday Fund, and ourselves—seven years ago. Since then certain minor differences in the method of applying the rules of the system have grown up, and during the year a joint committee of the three Funds has issued some supplementary regulations and suggestions. Mr. Griffiths, who was responsible for the original revision, again gave his expert aid as the representative of this Fund; and, again, as on the previous occasion, a committee of hospital secretaries assisted with their practical experience. In thus co-operating with the other two Funds we are not only doing what it is a real pleasure to do, but we are carrying out the intention expressed by our founder, of revered memory, in his original letter inaugurating the Fund.

Changes in the Personnel.

Several changes in the personnel of the Fund have to be recorded. We welcome the appointment on the Council during the year of the new Chief Rabbi. On the other hand we have to deplore three losses by death. Mr. Alfred Willett, though he had already retired from active service, had served for eleven years as a medical visitor and member of the Convalescent Homes Committee; Canon Barnett, first on the Distribution Committee and then on the Council, gave us so far as his health would permit, the benefit of his ripe experience and judgment in matters of charitable administration; and the Hon. Spencer Lyttelton had been a visitor for thirteen years. It is with great regret also that we view the approaching loss, through resignations, of three members of the Council—Sir Trevor Lawrence, formerly Vice-Chairman of the Distribution Committee, and Mr. J. Pierpont Morgan, both of whom have been connected with the Fund in various capacities since 1893; and Mr. J. Danvers Power, whose services as Honorary Secretary, member of Executive and Distribution Committees, and member of the Council, can hardly be over estimated.

Before I conclude may I, on behalf of the Governors, express our thanks to all the members of the Council, members of Committees, visitors, and honorary officers for their assistance during another successful year? Among the honorary officers this year must be included Mr. Fry, who very kindly resumed his old work at the office for a time during Sir Savile Crossley's absence abroad.

I now beg to move the adoption of the Reports of the Distribution and Convalescent Homes Committees.

The Speaker's Speech.

The Speaker of the House of Commons, in seconding the adoption of the Reports, said: "Your Highness, my Lords and Gentlemen,—I have much pleasure in seconding the motion which has been made by the Chair. There are three points which I think we ought to bear in mind as specially standing out in this year's Reports. First of all, the fact that we are able to maintain the high figure of £157,500 as the amount which we distribute. Secondly, the very munificent gifts which are just coming to us from Sir Julius Wernher's executors. In connection with this I might perhaps refer to the fact that it is only two years ago since the Fund received a large and generous gift from Sir Ernest Cassel which was then distributed. I do not know whether I may permit myself to express an opinion on this occasion in support of what fell from Lord Rothschild as to the advantage of not treating Sir Julius Wernher's gift upon the same footing as other legacies, but investing it and treating it as a permanent investment. It seems to me that that is a sound and what I may call a conservative policy to adopt.

Last of all, may I say a word as to the satisfaction with which I have observed that the slightly strained relations which existed between ourselves and St. George's Hospital have, we may trust, come to an end? I should hope for myself that we may now "bury the hatchet," if ever any hatchet was employed at all, and that our relations may no longer be strained, but may be of the cordial character that they were before this unfortunate and unhappy dispute arose. I have very great pleasure in seconding the resolution.

After discussion the adoption of the Reports was then put and carried.

On the motion of the Chief Rabbi (the Rev. Dr. Hertz), seconded by Sir Vezey Strong, it was resolved "That the cheques for the grants should be posted on December 18 except in the case of grants for the reservation of beds in consumption sanatoria during 1914, which should be posted on January 1."

On the motion of Mr. Sydney, seconded by Sir John Tweedy, a report of the Executive Committee on a resolution received from the Society for the State Registration of Trained Nurses was adopted.

Arrangements for 1914.

His Highness the Duke of Teck then said: My Lords and Gentlemen,—With regard to the arrangements for 1914, I have to announce that the Governors intend to appoint the members of the General Council, Standing Committees, and honorary officers in accordance with the list which I will now ask the Honorary Secretaries to read.

The list having been read by Sir Savile Crossley (Hon. Secretary), his Highness continued: We have now to consider the delegation resolutions, so that the meeting held early in January for the purpose of passing them need only be formal. All the resolutions are in the same form as last year.

On the motion of the President of the Royal College of Physicians (Sir Thomas Barlow, Bart.), seconded by Sir Horace Marshall, a resolution was approved delegating powers to the Finance Committee, Distribution Committee, and Convalescent Homes Committee.

On the motion of the Governor of the Bank of England (Mr. Walter Cunliffe), seconded by the Rev. Dr. W. L. Watkinson, a resolution was approved delegating powers to the Executive Committee.

The Lord Mayor (Sir T. Vansittart Bowater) proposed a vote of thanks to his Highness the Duke of Teck for presiding. The resolution was seconded by the President of the Royal College of Surgeons (Sir Rickman J. Godlee, Bart.), and carried unanimously.

The Duke of Teck having briefly acknowledged the vote of thanks, the proceedings then terminated.

